# The connection between stress, density, and speed in crowds

**DOI:** 10.1038/s41598-023-39006-8

**Published:** 2023-08-21

**Authors:** Mira Beermann, Anna Sieben

**Affiliations:** 1 Fakultät für Sozialwissenschaft, Lehrstuhl für Sozialtheorie und Sozialpsychologie, Ruhr-Universität Bochum, Universitätsstr. 150, Gebäude GD E1.259, Postfach 78, 44801 Bochum, Germany; 2 Civil Safety Research (IAS-7), Institute for Advanced Simulation, Forschungszentrum Jülich GmbH, Wilhelm-Johnen-Straße, 52425 Jülich, Germany; 3 Department Psychology, School of Humanities and Social Sciences, Universität St Gallen, Dufourstrasse 50, 9000 St. Gallen, Schweiz

**Keywords:** Physiology, Psychology

## Abstract

Moving around in crowds is part of our daily lives, and we are used to the associated restriction of mobility. Nevertheless, little is known about how individuals experience these limitations. Such knowledge would, however, help to predict behavior, assess crowding, and improve measures for safety and comfort. To address this research gap, we conducted two studies on how constrained mobility affects physiological arousal as measured by mobile electrodermal activity (EDA) sensors. In study 1, we constrained walking speed by externally imposing a specific walking speed without physical proximity to another person, while, in study 2, we varied walking speed by increasing the number of people in a given area. In study 1, we confirmed previous findings showing that faster speeds led to statistically significantly higher levels of physiological arousal. The external limitations of walking speed, however, even if perceived as uncomfortable, did not increase physiological arousal. In the second study, subjects’ speed was gradually reduced by density in a single-lane experiment. This study shows that physiological arousal increased statistically significant with increasing density and decreasing speed, suggesting that people experience more stress when their movement is restricted by proximity to others. The result of study 2 is even more significant given the results of study 1: When there are no other people around, arousal increases with walking speed due to the physiology of walking. This effect reverses when the speed must be reduced due to other people. Then the arousal increases at lower speeds.

## Introduction

Pedestrian dynamics is an interdisciplinary field of research that draws on mathematics, physics, computer science, and psychology, connecting concepts from each in the interest of better understanding human behavior in crowds^1^. Over the last two decades, research into pedestrian dynamics has increasingly incorporated the social sciences to deal with questions of social group status and anonymous crowd behavior, but also with the individual experiences of people in crowds and pedestrian environments^2–14^. This approach is important to complement and improve concepts based on the principles of physics and computer simulations.

One of the most basic factors in the analysis of pedestrian dynamics is the relationship between density and speed (velocity, flow). This is depicted in what is referred to as the fundamental diagram^15^—similar to vehicular traffic. Although it is well described and understood how increasing density reduces speed, how people experience this remains an open question. The general assumption is that it is more stressful to be forced to walk slowly in high density situations. In a quantitative study of Jia and colleague^10^, participants indeed reported negative experiences with increased proximity to others and reduced speed. To our knowledge, however, this has not thus far been quantitatively investigated in any detail.

Nonetheless, the assumed connection between density and stress plays an important role in the evaluation of pedestrian infrastructure, first and foremost in the engineering concepts called pedestrian level of service (PLOS)^16^. PLOS concepts are a common way to describe quality and comfort, and thus safety in regard to pedestrian infrastructure. This approach uses density and flow velocity to evaluate crowds and crowd comfort.

A number of PLOS concepts have evolved since 1911^17^. They have been developed to describe the quality level and functionality and comfort of pedestrian infrastructure, and they are used in planning and designing facilities, sidewalks, stations, and theaters^17–21^. Scholz^22^, for example, deals with the speed of walking and the design of pedestrian facilities. In his work, he assumes that a person’s natural gait is between 0.5 and 2.0 m/s. All speeds below 0.5 m/s increase the effort required to maintain balance. Oeding^23^ took up the concept of natural gait. For him, the speed of the normal gait was between 0.5 and 1.8 m/s. In addition, he investigated the relationship between walking speed and traffic density and, based on these investigations, formed five density-dependent quality levels of pedestrian walking. Subsequent concepts developed used density as an indicator of quality. For example, Fruin^18^ established a six-level assessment system, while Weidmann^21^ developed a nine-level system. In addition to density, Weidmann’s assessment of quality levels was based on criteria he extracted from a literature review of two hundred walking studies. His quality criteria are:

1. Possibility of free speed selection
2. Frequency of a forced speed change
3. Compulsion of attention to other pedestrians
4. Frequency of forced changes in direction
5. Restrictions with opposite direction of movement
6. Restrictions on overtaking
7. Frequency of unintended physical contacts

All these criteria increase as density increases in traffic infrastructure, leading to more negative experiences of the infrastructure. Similarly, Filingeri and colleagues^9^ found in their qualitative study of participants’ views of crowd experiments that movement in crowds affects participants’ enjoyment and satisfaction. They stated that having one’s movement restricted by other people and unwanted proximity are common negative experiences in crowds.

**Figure 1:**
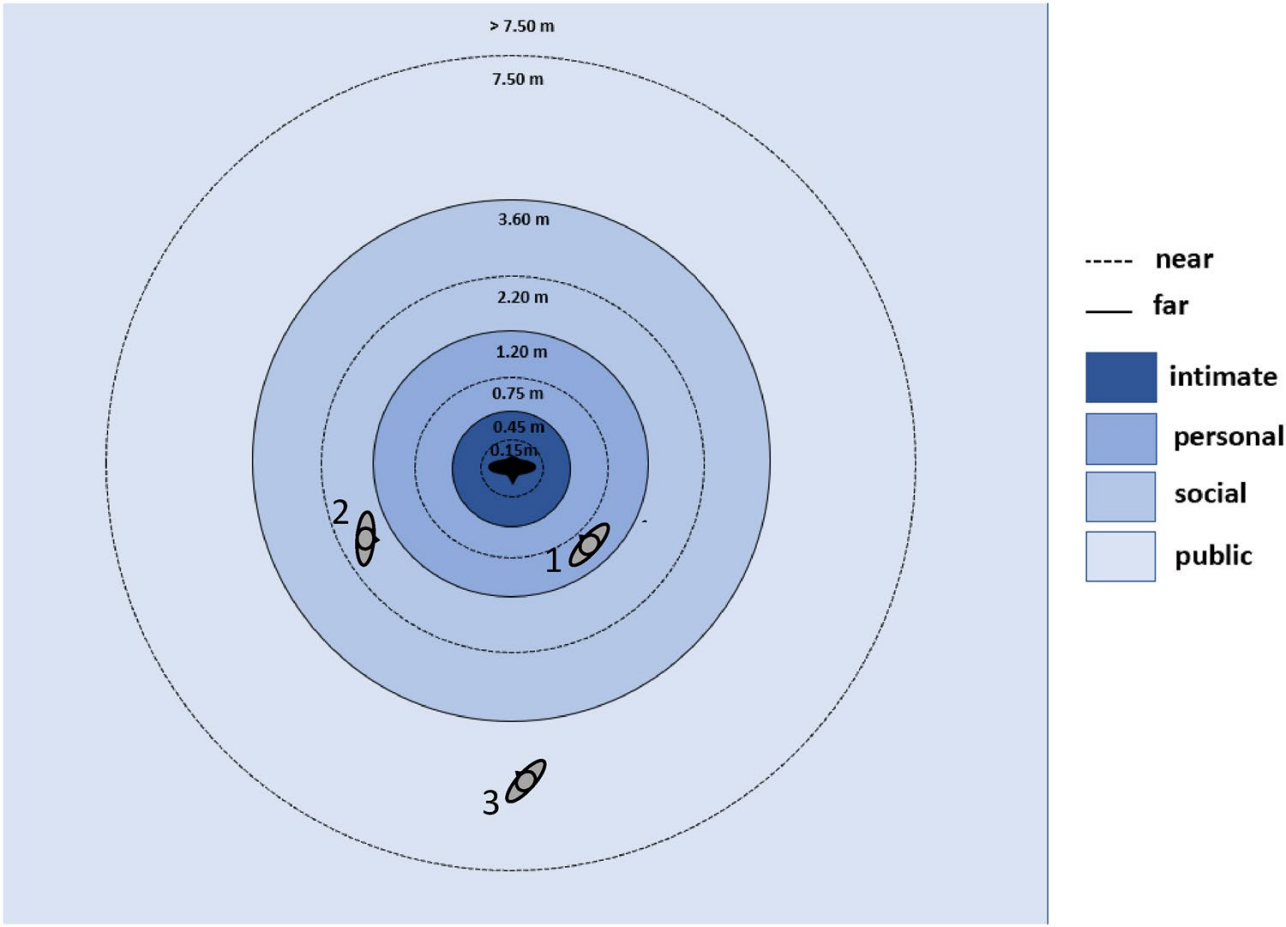
Distance zones according to Hall^24^ with strangers exemplifying the violation of the personal far zone (1), the social near zone (2) and the public far zone. The darker the color of a zone, the more unpleasantly the violation by a stranger is perceived^25^.

Unwanted proximity to other people in crowds is a factor in Fruin’s study of the PLOS concept^18^. He adds a “personal body buffer zone concept” to PLOS, based on the concept of proximity by Hall^24^. According to Hall, the space around a person can be divided into four different zones, into which different people are allowed to enter depending on their relationship and the situation (Fig. 1). Violations of these rules lead to stress^25^. Each of the four zones is subdivided into a near and a far zone.

Researchers other than Fruin have likewise integrated this criterion into their theoretical concepts^16^. As outlined above, however, only rarely have scholars applied psychological methods to investigate these experiences of distance and density.

Because the term “experience” is rather broad, this study focuses on one particular aspect of experience, namely stress. Stress is the non-specific reaction of the organism to any kind of disturbance of homeostasis and one must distinguish stressors (stimuli) from stress (reaction)^26^. This concept fits well into the PLOS perspective on the safety and comfort of pedestrian infrastructure. One way to operationalize stress is to measure its physiological correlates such as arousal^26^. A well-established method in psychology for recording arousal is the measurement of the change in skin conductance by electrodermal activity (EDA)^27–29^. Here, EDA is measured in terms of the change in activity of the eccrine sweat glands, which are controlled by the sympathetic nervous system. Thus, EDA indirectly measures changes in sympathetic nervous system activity. The sympathetic nervous system responds to threatening and stressful situations with higher activity to ensure survival—the so-called fight-or-flight response^30^. When the sympathetic nervous system is activated, skin conductivity increases, which means that it can be used as an indicator of arousal^27^. The indication of physiological arousal measured with EDA, however, does not distinguish between different qualities of stress, even though subjective experiences of stress can be both positive or negative^31^. The development of smaller, portable EDA recording devices has allowed for this method to be expanded in field research^11,32–35^.

Measuring physiological arousal with EDA is increasingly finding applications in pedestrian dynamics research (^36–40^, see^41^ for an overview). One of the first empirical studies on the “personal buffer comfort zone” using EDA was conducted by Engelniederhammer and colleagues^36^. In their study on the relationship between well-being and violations of personal space, subjects in Hong Kong were sent on different street routes and the stress response, measured using EDA, was examined. Subjects’ personal space was recorded using a chest sensor that detected any intrusion into the area 2 m in front of the subjects. The study found that violations of personal space resulted in increased stress levels. In their walking study of the perception of different environments, while walking, LaJeunesse and colleagues^37^ found increased stress levels in areas associated with increased density. These include, for example, streets with many office buildings and stores for shopping. These studies did not investigate density and associated personal space violations. The two studies presented suggest that personal space violations lead to higher stress responses. However, Beermann and Sieben^40^ showed that higher densities do not necessarily lead to higher stress levels. They studied the effect of density on stress levels in a stationary setting by placing 2, 4, 6, or 8 participants in a “tiny box” consisting of 1 m^2^. Stress levels were shown to be higher at densities of 2 persons/m^2^ than at densities of 4 and 6 persons/m^2^. In addition, there was a continuous adaptation of stress levels—a decrease in the EDA level—over time. The questionnaire revealed that the densities of 6 and 8 people were perceived as the most uncomfortable, although the density of 2 people was also perceived as unpleasant. Overall, the study showed that parameters other than density obviously influence stress levels while waiting. Beermann and Sieben’s results^23^ raise the question of whether there is a relationship between the violation of personal space and the decrease in the quality of experience in high density situations for humans.

In contrast to the “tiny box” experiment which consisted of a waiting scenario, the studies presented here are interested in the relationship between walking speed, density, and stress measured in terms of arousal. Density may play a different role in waiting and walking. When walking, people are involved in a complex process of body movement to remain balanced, to adapt to the physical environment, to steer, and to find the right speed for the context. When a crowd situation becomes denser, people must adapt to others around them, and they might not be as fast as desired. Therefore, reduced speed in a crowd might increase stress both because it creates a motivational gap (between actual and desired speed) and because of the extra cognitive load. “Cognitive load” is a term from Chandler and Schweller’s^42^ learning theory and refers to the workload of working memory during learning. Working memory is responsible for problem solving and information processing. Thus, working memory is responsible for such processes as way-finding and orientation, but also responsible for paying attention to others. A study by Armougum and colleagues^43^ shows that working memory activity is also associated with increased physiological response while way-finding. Furthermore, walking in a dense crowd might also increase concerns of being pushed or hurt by others (for example, if they step on your feet). A recent treadmill study from Ogden and colleagues^38^ looked at the influence of environmental conditions on EDA or stress levels. This study used virtual reality. The study design included videos of different environments—indoor, outdoor, or green environments. Using videos, crowded situations and empty situations were projected over the different environmental representations. The conditions with crowded videos showed higher EDA values in the study. Unfortunately, no data on walking speed was available for the study. There was also no description as to whether the speed was constant. Thus, no conclusions could be drawn about the influence of speed and any associated moderating effect.

When using EDA measurements while participants are walking, some methodological aspects need to be considered that have not been given much attention in the field of pedestrian dynamics so far. Potentially, walking can impair the measurement of arousal in two ways, first by creating motion artifacts (i.e., sensors lose contact), second by increasing the physiological activity of the body. There are studies dealing with artifacts in EDA due to walking^44,45^, but these are more about automated detection of artifacts and not about the influence of walking on arousal. In addition, there are only a few studies describing the influence of walking on arousal. For example, Schumm and colleagues^46^ describe the changes in EDA observed in their studies with walking, slow running, and fast running. In the study, they found increased EDA levels with higher physical exertion due to faster running. Possada, Reljin, and Mills^47^, in their treadmill study, showed an increase in EDA as a function of speed when speed was varied from walking at 4.82 km/h to fast running at 8 km/h and even faster. He assumed that additional artifacts due to motion could be excluded due to the design used. This study suggested an influence due to speed was possibly moderated by perspiration.

As a result of the limitations outlined, the studies described do not answer conclusively how the effects of speed and density as well as personal space, which all act simultaneously, are related to the arousal recorded via the EDA. To broaden this view and gain a better understanding of the crowd experience, this paper uses two psychological experiments to analyze the experience of pedestrians in crowds of varying density. For this purpose, two studies were conducted, one examining the effect of walking speed on stress parameters and the other looking at the effect of walking speed limited by density. To measure psychological experience—stress and arousal more specifically—a physiological methodological approach was chosen in the studies described below. EDA procedures were used to record psychological reactions. This allowed us not only to compare the reactions to different experimental conditions in quantitative terms, but also to record their timeline during the entire experiment. The studies presented below take these influencing factors into account in a two-study design and thus contribute to the further development of theory. Two studies were designed to distinguish the relationship between walking speed and stress from the relationship between density-dependent walking speed and stress. The first study examined the effect of predetermined changes in walking speed from “normal speed” to significantly slower walking speeds. In addition, it also addressed the effect of a freely chosen speed compared to having the speed imposed externally, as this is a key feature of the criteria in PLOS. In the second study, we investigated the relationship between density and speed by using a well-established experimental setting in pedestrian research, namely a situation in which more or fewer participants walk in a defined oval^48^.

This experiment is usually used to measure the fundamental diagram^48^—which is also a basis for the PLOS. This diagram shows the relationship between density and walking speed. Weidmann^21^ used density to assign certain sections of the graph to his comfort classes in the PLOS already mentioned above. To investigate this correlation empirically, Seyfried and colleagues^48^ developed a simplified experiment which only investigated the correlation between walking speed and density, while disregarding all other aspects of Weidmann’s concept like the possibility to overtake. In the experiment setup, an oval was drawn on the floor, and the test subjects were to walk along the oval one behind the other in a line. More and more people were added to the line, so the oval became increasingly full. This increased the density and speed decreased. In this experiment, Seyfried and colleagues^48^ found that the results on the relationship between speed and density from the experimental study for single-lane movements and the values from Weidmann’s study were consistent. Thus, Seyfried and colleagues^48^ created a possibility to investigate the complex relationship between density and speed specifically and to investigate the influence of external factors such as gender and culture on the relationship. This experimental setup also allowed us in our second study to look at the effect of the relationship between speed and density on stress measured by physiological arousal by adding EDA sensors.

## Study 1

### Hypotheses

1. Walking with a freely chosen walking speed is less stressful and therefore shows lower skin conductivity parameters than externally imposed walking speeds.
2. Forcing slower walking by externally imposed walking speeds leads to an increase in skin conductance parameters.
3. Individuals who want to overtake have higher stress values in the slower conditions and thus have higher values in the skin conductance parameter.
4. Individuals who normally walk fast are more stressed in the slower walking conditions and thus have higher values in the skin conductance parameter.

## Results

### Result: questionnaire

The means and variances of the individual questions from the questionnaire collected at the end of the experiment can be found in Table 1. To investigate the influence of the freely chosen speed on the perception of the experiment, we divided the sample into two groups (fast and slow, see above). We found that there was no significant difference between the assessments on the perception of the very slow speed and the desire to overtake. Nevertheless, the trend of the mean values of the groups indicates that those who chose to walk slowly when they were walking alone reported a greater desire to overtake and a more uncomfortable feeling when walking slowly in the group (see Table 1). This effect is counterintuitive: We would have expected that those who had a more audible free walking speed would also have a stronger need to overtake and would have been more annoyed by the slow speed. The effect sizes of the test indicated a moderate effect (Cohen’s d = 0.57). The individuals who find the slowest condition most uncomfortable stated that they found it difficult to maintain balance and consider walking slowly a waste of time.

**Table 1:** Table showing the questions of the questionnaire with the corresponding means and standard deviations (in brackets).

	Question	Overall mean	Group mean fast	Group mean slow
1	I felt observed over the course of the experiment by other people (not part of the experiment)	2.06 (1.09)	2.00 (1.00)	2.13 (1.25)
2	I felt the need to overtake the person in front of me	2.88 (1.58)	2.44 (1.74)	3.38 (1.3)
3	The very slow speed was uncomfortable for me	3.47 (1.12)	3.11 (1.45)	3.88 (0.35)

### Results: skin conductance

**Figure 2:**
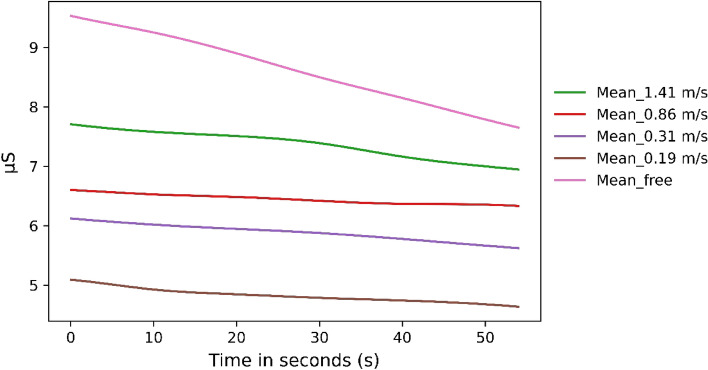
The averaged SCL over time across all subjects for the time of the freely chosen walking speed.

When qualitatively examining the SCL data over time, it is noticeable that the level of arousal at the freely chosen speed was significantly higher than those at the externally imposed speeds. In addition, there was a clear separation between all conditions (Fig. 2). Looking at the mean SCL values, we found some difference (Friedman-Test: Χ^2^ (4) = 38.17, *p* < 0.001) over time (Tables 2 and 3).

**Table 2:** Significant difference for the EDA parameters with *p* values for the time period of the freely chosen walking speed.

	Mean_SCL	EDASymp	Amplitude
Free chosen speed—0.89 m/s	*p* = .007	*p* = .015	*p* = .006
Free chosen speed—0.31 m/s	*p* = .006		
Free chosen speed—0.19 m/s	*p* = .005		*p* = .05
1.41–0.31 m/s	*p* = .033		
1.41–0.19 m/s	*p* = .003		*p* = .05

**Table 3:** Means and standard deviations in brackets for all conditions of the first 54 s to be able to compare the values of the preset speed to the values of the free chosen speed.

	Freely chosen Speed	1.41 m/s	0.86 m/s	0.31 m/s	0.19 m/s	Baseline
Mean_SCL	8.54 µS (3.91 µS)	7.31 µS (3.58 µS)	6.43 µS (2.91 µS)	5.87 µS (3.12 µS)	4.81 µS (3.42 µS)	6.55 µS (3.18 µS)
EDASymp	.038 (.048)	.02 (.027)	.01 (.016)	.015 (.027)	.0098 (.018)	.016 (.025)
Amplitude	.0022 µS (.0021 µS)	.0016 µS (.0017 µS)	.00074 µS (.00066 µS)	.00073 µS (.00095 µS)	.014 µS (.001 µS)	.001 µS (.002 µS)

The amplitude of NS.SCR also differed significantly between the free-choice velocity and slow conditions (Friedman-Test: Χ^2^ (4) = 26.02, *p* < 0.001). Looking at the EDASymp parameter, we found a significant difference between the conditions “freely chosen walking speed” (Friedman-Test: Χ^2^ (4) = 20.61.17, *p* < 0.001) and the given mean slow condition of 0.31 m/s. Significant differences were not found between any conditions in the comparisons of Ns.SCRs.

**Figure 3:**
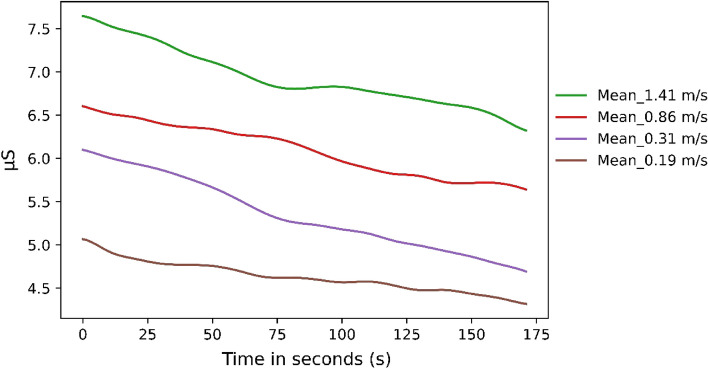
The averaged SCL over time across all subjects over the experimental run-time of 3 min per condition.

In addition to comparing the freely selected walking speed with the externally imposed walking speeds, the times of the externally imposed walking speeds were also compared with each other. The quantitative analysis reveals that the averaged SCL values decreased over the time of the experimental conditions for all experimental conditions (Fig. 3). The graphs do not intersect or show different excitation levels. It should be noted that the faster the subjects walked, the higher the graphs of the stress values. This trend is also evident in the quantitative examination of the parameters. Here, significant differences in SCL values are found (Friedman-Test: Χ^2^ (3) = 29.54, *p* < 0.001) (Tables 4 and 5). The amplitudes of Ns.SCRs also differ significantly (Friedman-Test: Χ^2^ (3) = 15.54, *p* < 0.002). Sympathetic activation measured by the parameter EDASymp was significantly different between the conditions (Friedman-Test: Χ^2^ (3) = 14.51, *p* = 0.014). The last two comparisons show no significant difference in the post hoc tests.

**Table 4:** Significant difference between the conditions for the EDA parameters with *p* values over the experimental run-time of 3 min per condition.

	Mean_SCL
1.41–0.86 m/s	*p* = .007
1.41–0.31 m/s	*p* = .002
1.41–0.19 m/s	*p* = .005

**Table 5:** Means and standard deviations in brackets for all conditions and all EDA parameters over the experimental run-time of 3 min per condition.

	1.41 m/s	0.86 m/s	0.31 m/s	0.19 m/s	Baseline
Mean_SCL	6.91 µS (3.1 µS)	6.08 µS (2.68 µS)	5.33 µS (3.05 µS)	4.62 µS (3.32 µS)	4.23 µS (2.95 µS)
EDASymp	.023 (.026)	.013 (.021)	.018 (.032)	.016 (.024)	.014 (.025)
Amplitude	.0013 µS (.0013 µS)	.00067 µS (.0007 µS)	.00073 µS (.00095 µS)	.0065 µS (.00091 µS)	.001 µS (.002µS)

### Influence of habitual walking speed and the desire to overtake

The influence the person’s typical walking speed had on their desire to overtake others was investigated statistically with the help of mixed models. To ensure a comparison between the groups, the EDA data were standardized. However, it turns out that neither the freely chosen speed nor the desire to overtake had a mediating influence on the EDA parameters. The model parameters for the SCL are in Tables 6 and 7, the other model parameters are in the supplementary material.

**Table 6:** The model for the standardized SCL and the mediator “desire to overtake”.

Parameter	β	t value	*p*
Intercept	−8.64	5.34	< .001
Speed	−.80	−2.97	.00599
Desire to overtake	−.34	−.688	.502
Speed: desire to overtake	.014	−.168	.867

**Table 7:** The model for the standardized SCL and the mediator “slow freely chosen walking speed”.

Parameter	β	t value	*p*
Intercept	7.81	6.98	.001
Speed	−.78	−4.23	.001
Slow freely chosen walking speed	−.28	−.18	.858
Speed: slow freely chosen walking speed	.04	.14	.888

## Discussion

The results of the study show two interesting aspects: First, the comparisons of the varying walking speeds show that fast walking is associated with increased stress levels. This is evident in several parameters. Although this effect is contrary to our hypothesis, the increased response can be explained by the activation of the sympathetic nervous system due to the physical activity. For example, Posada-Quintero and colleagues^47^ showed in an exercise study that walking at increased speeds on a treadmill was associated with increased sympathetic activity and thus higher EDA levels. The assumption that changes in walking speed from the natural gait to low walking speeds are perceived as more difficult and unpleasant was not confirmed by the study. Nevertheless, the questionnaire results do include statements such as “Because it was much slower than I was used to and I had a hard time coordinating my body (legs & feet),” which support the idea that an increase in cognitive effort is required to maintain balance. Nevertheless, the results showed that this effort affected stress levels and sympathetic nervous system activity less than physical activation did in the fast conditions.

Furthermore, it is seen that a freely chosen speed of 1.39 m/s on average is more arousing than walking behind the experimenter according to externally imposed speeds. These results are not only evident in the qualitative observation, but also in the quantitative comparisons. Possible explanations for this higher state of arousal include that more higher cognitive load is associated with walking alone at free speed, such as way-finding and orientation, whereas these processes are not needed while following a person. Two studies by Amougum and colleagues^43,49^ show increased physiological responses when using cognitive processes in real-life travel experience and also in virtual reality travel experience. Additionally, people walking alone might have felt more insecure about whether they were performing the experiment “correctly” and might have felt observed by the experimenter.

The study is limited by the fact that it is a pure laboratory study, and the results cannot be transferred one-to-one to real life. For example, the individuals in the study were not intrinsically motivated to be fast and did not have deadline pressure as they might in real-life situations. Nevertheless, the study provides some insight into the physiology of walking and allows for further studies to add other parameters such as motivation or deadline pressure to investigate the impact of other internal and external stressors.

Overall, the results of this study indicate that the free choice of walking speed alone does not result in a low-stress experience in crowds. Rather, it should be considered that a freely chosen walking speed involves cognitive and social processes that may lead to increased arousal states^49^. Therefore, it may be more pleasant to walk in a stream of people with an adapted walking speed and the same spatial destination than to find one’s way. In addition, and most importantly, it is shown that physical exertion has a significant effect on skin conductance. This must be considered in further studies involving walking. All these results should always be interpreted with the information that the distance to the person in front was kept constant, and thus no influence of a personal space violation needs to be considered as an influencing variable. The PLOS, however, as explained above, includes this information. To systematically consider the influence of the violation of personal space, we carried out the second study. For this purpose, we reduced the walking speed by increasing density and then measured the stress parameters again.

## Study 2

### Hypothesis

1. The slower the subjects walk because of increasing density, the more stressed they are and thus the higher the values in the skin conductance parameter.

## Results

**Figure 4:**
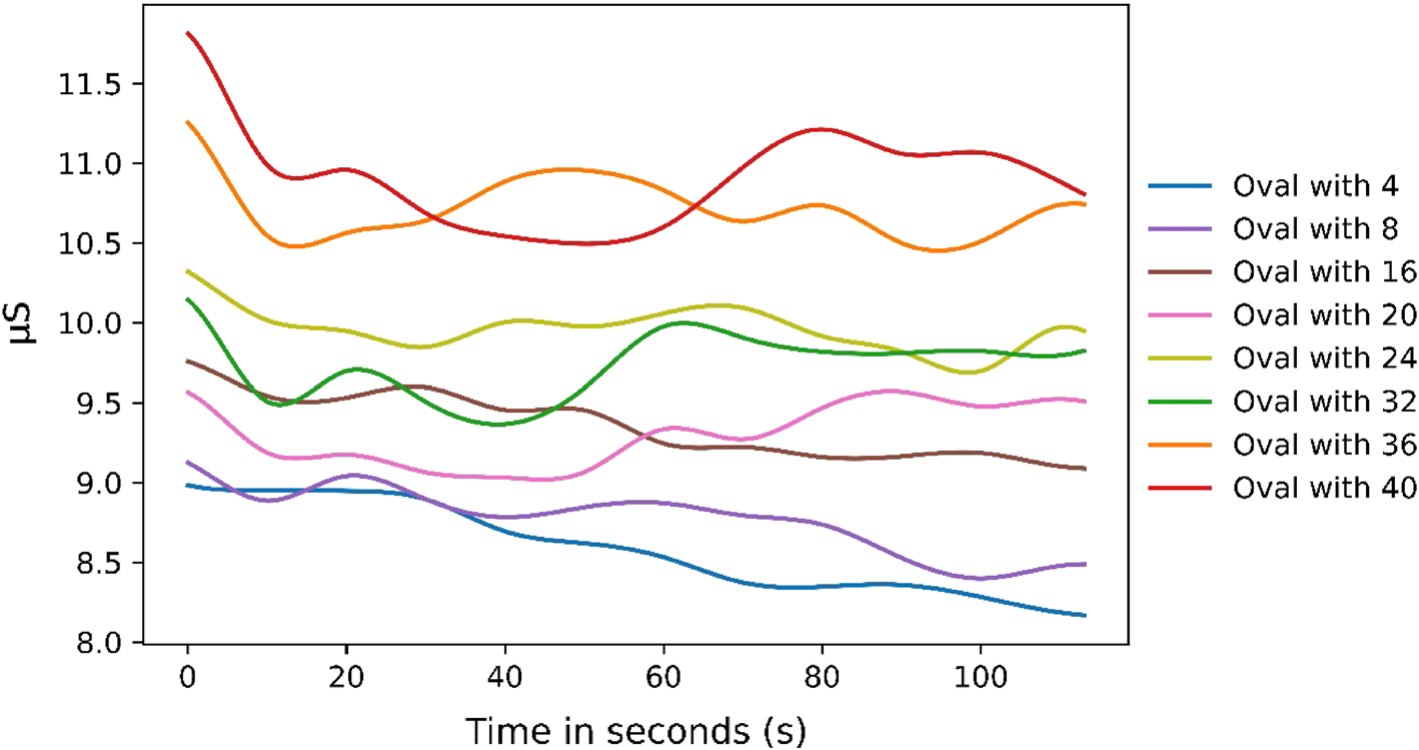
The averaged SCL over time across all subjects.

Looking at the EDA’s SCL data over time, it is noticeable that there is a sorting by the different conditions. It is noticeable that the graphs of people in the conditions with 4 and 8 people are close together and are the least stressful. This is also true for the graphs of conditions with 36 and 40 persons. The graphs of conditions with 16, 20, 24, and 32 persons are unsorted and close to each other in the middle range. Here, at the end of the time, a little less than 2 min, one sees a sorting of the graphs according to the conditions. It goes from low densities and low stress values to high densities and high stress values (see Fig. 4). In addition, it can be seen that there is an adaptation phase in which all graphs, except the one when there are 4 people in the oval, fall (0–20 s). After that, the graphs fall only slightly and a few rise again.

Furthermore, looking at the distribution, the SCL value increases as the density increases (Fig. 4). When looking at the quantitative results, several significant differences (F (3.88;166.70) = 10.73, *p* < 0.001, Greenhouse–Geisser corrected) emerge for the parameter SCL (see Tables 8 and 9).

**Table 8:** Significant difference for the EDA parameters with *p* values.

Contrast	Mean_SCL	EdaSymp	Amplitude	NS.SCR
4 Subjects–24 Subjects	*p* = .033			
4 Subjects–32 Subjects			*p* = .005	
4 Subjects–36 Subjects	*p* = .001	*p* = .032	*p* < .001	
4 Subjects–40 Subjects	*p* < .001		*p* = .003	
8 Subjects–32 Subjects			*p* = .034	
8 Subjects–36 Subjects	*p* < .001		*p* = .001	*p* = .012
8 Subjects–40 Subjects	*p* < .001		*p* = .020	*p* = .026
16 Subjects–32 Subjects			*p* = .040	
16 Subjects–36 Subjects	*p* = .023		*p* = .007	
16 Subjects–40 Subjects	*p* = .019			
20 Subjects–36 Subjects	*p* = .008		*p* = .003	
20 Subjects–40 Subjects	*p* = .010			
32 Subjects–36 Subjects	*p* = .023			
32 Subjects–40 Subjects	*p* = .048

**Table 9:** Means and standard deviations in brackets for all conditions.

Condition	Mean_SCL	EDASymp	Amplitude	NS.SCR
4 Subjects	8.58 µS (3.87 µS)	.04 (.07)	.0015 µS (.0012 µS)	18.14 (6.9)
8 Subjects	8.77 µS (4.39µS)	.07 (.14)	.0017 µS (.0018 µS)	16.52 (6.26)
16 Subjects	9.35 µS (5.00 µS)	.05 (.06)	.0018 µS (.0018 µS)	16.66 (7.54)
20 Subjects	9.3 µS (5.29 µS)	.1 (.25)	.0020 µS (.0020 µS)	17.91 (9.69)
24 Subjects	9.95 µS (5.33 µS)	.11 (.2)	.0024 µS (.0027 µS)	18.43 (8.29)
32 Subjects	9.72 µS (5.05 µS)	.13 (.22)	.0027 µS (.0025 µS)	17.48 (7.09)
36 Subjects	10.71 µS (4.98 µS)	.17 (.26)	.0032 µS (.0027 µS)	19.5 (6.4)
40 Subjects	10.9 µS (4.93 µS)	.19 (.35)	.0032 µS (.0031 µS)	19.7 (6.7)

The amplitude shows some significant differences between the conditions (F (3.99;171.48) = 9.14, *p* < 0.001, Greenhouse–Geisser corrected) (Tables 8 and 9). Moreover, the sympathetic activation parameter shows significant higher values (F (4.09;175.84) = 3.76, *p* = 0.006, Greenhouse–Geisser corrected) (Tables 8 and 9). Also the comparisons of Ns.SCRs find a significant difference (F (4.32;185.67) = 2.49, *p* = 0.041, Greenhouse–Geisser corrected).

## Discussion

This study shows that density has a significant effect on sympathetic nervous system arousal while walking. The different parameters (SCL, EDASymp, amplitude) of EDA show an increase of stress with increasing density. In agreement with Engelniederhammer and colleagues^36^, this experiment suggests that the violation of personal space leads to increased stress levels. However, our consideration of personal space is more nuanced than that found in Engelniederhammer and colleagues^36^ and reveals differences between the individual distance zones in this regard. The degrees to which the oval was filled in the study can be assigned to the different distance zones according to Hall^24^ based on the space available in the individual conditions. Thus, the condition with 4 persons corresponds to the far social distance zone and the condition with 8 persons to the near social distance zone. The conditions 16 persons and 20 persons can be assigned to the personal near distance zone. The conditions with 24 and 32 persons belong to the intimate far distance zone. The conditions with 36 persons and with 40 persons belong to the intimate close distance zone.

The results of the study show that the social far distance zone and personal near distance zone lead to less stress than the intimate near distance zone. The personal far condition was not surveyed in this study but, based on the results collected, would probably also differ from the intimate near zone conditions. Overall, it appears that reduced space leads to more stress, and this then leads to an increase in the parameters.

When looking at the graphs of the 4 and 8 person conditions, it is noticeable that they are close to each other. Although they belong to the same distance zone, there is a difference in available space between them, and they are in different subcategories: one is close and the other is far. However, the lack of difference between the 4 and 8 person conditions shows how big the difference is between this zone and others in terms of stress level. It can be concluded that the social distance zone does not generate a higher level of stress compared to the other zones. It can also be seen that no significant differences can be measured within these individual distance zones. This calls into question the distinction between near and far zones in walking in terms of the stress level triggered. Further studies are needed here to obtain a more comprehensive picture. Besides this aspect, it is noticeable that all graphs except for the one with four people in the oval sink at the beginning. A possible explanation for this would be a habituation effect to the proximity to other people, since this feature can also be found in the data of the 'tiny box' by Beermann and Sieben^40^. This habituation is not necessary with four people in the oval, because there is no violation of personal space. Another possible explanation is that in this condition a high walking speed can be maintained so that arousal does not decrease due to physical activity.

The study shows the unsurprising observation that moving in a confined space leads to increased stress experience. Unlike the study by Beermann and Sieben^40^, which found no correlation between stress experience and waiting in varying dense situations, it can be said here that increasing density and thus a reduced amount of space leads to increased stress levels in moving people. One possible explanation for the results arises from the additional cognitive effort required when moving. Slow walking requires more attention when trying not to touch the person in front of you and having to match their rhythm. Furthermore, walking in a very dense crowd always carries the risk of pressure or being hurt/hurting someone, while the risk of sudden pressure is very low in studies with stationary settings like the Tiny Box^40^.

The results of study 2 are also consistent with the assumptions that quality of walking for pedestrians decreases as density increases. Thus, this very reduced-scale experiment suffices to show that changes in the distance to the person in front and behind influence stress scores. It did not consider the presence of people to the left and right while walking, which would further increase density. Moreover, this experiment leaves open the question of what influences Weidmann’s criteria, such as speed change or time pressure, have on the stress level. It would also have been interesting to survey subjective perceptions after each experimental condition to compare subjective values with objective values. Furthermore, future studies should address the smaller densities leading to violations of the distance range “personal far,” which unfortunately, was not done in this study.

The two studies complement each other: Whereas in the first study arousal was higher when walking faster, the opposite was measured in the second study. From our perspective, this underlines the strong influence of stress due to increased density and proximity in the second study. Given that walking slower requires less body activation, it is significant that walking slower in high density environments produces higher arousal than walking faster in low density situations. The combination of study 1 and 2 allows for differentiating the effects of walking speed by itself and the effects of walking speed reduced by density on physiological arousal. However, it should be noted that Study 1 and 2 were not conducted with the same participants in the same setting. Even more precise results can be expected if the studies are conducted with the same individuals immediately one after the other.

Furthermore, these studies have shown that data loss is mainly due to the generation of zero lines by the loss of electrode contacts. Additionally, the detection of artifacts using Gashi et al.'s method^45^, which targets motion artifacts, reveals that rule violations can also be present in the baseline survey of study 1, where participants remain still with no physical activity detected. As a result, a systematic examination of the rules should be conducted. The tests should be reviewed to ensure accuracy in further studies to enable use in the field. However, it must be taken into account that the subjects were instructed not to move their hands excessively. In addition, the experiment periods were short, less than 45 min, and the velocities were moderate to slow and were controlled. An automatic derivation of the results for surveys in the field is therefore difficult. So, the subjects were not exposed to unintentional contact, such as jostling, in the dense situations, for example.

The two studies demonstrate the influence of a) personal space violation and b) speed on stress levels while walking. Thus, they provide a better understanding of the stress experience of people in crowds: Contrary to situations in which participants are waiting, density matters when walking. Increased density (decreased personal space) goes along with higher arousal. Furthermore, from a methodological perspective, Studies 1 and 2 further our understanding of the physiology of walking and its influence on the EDA. The walking speed indeed increases arousal as measured with EDA. This needs to be considered when interpretating EDA data in walking experiments. This was rather easy in the case of study 2 because the results were in the opposite direction and therefore could be interpreted as an effect of stress. The experiments thus show that EDA is a good method for studying sympathetic arousal in pedestrian dynamics research. The method opens numerous possibilities for future studies to get a better impression of how people feel in crowds and which factors have an influence on lowering quality in pedestrian infrastructure.

## Study 1

### Method

#### Participants

Subjects were recruited through announcements in university lectures, Facebook university groups, and notice boards. After checking the EDA signals for artifacts such as null lines or extreme fluctuations due to movement or contact problems of the electrodes, we excluded 8 participants from further analysis. The sample includes a total of 17 subjects. This was a student study, and the average age of the participants was 27.59 years. Of the 17 subjects, 12 (71%) were female and 5 (29%) were male. We assume that gender might influence the experience in dense crowds. However, in this experimental setting, all subjects were kept at a distance of 1.5 m. Therefore, in this experiment we did not expect any effects of gender and decided to not control for the gender distribution when conducting the experiments. Each subject received €10 for participation. Each of the subjects signed an informed consent form after being informed of the experimental procedure. All methods were performed following the Declaration of Helsinki.

#### Procedure and experimental paradigm

The study was conducted at the Ruhr University in Bochum, Germany. Due to the Covid-19 pandemic, experiments with crowds were difficult, so the experiments were conducted with a maximum of five subjects at a time and outside. Masks were worn by the subjects in all runs. Subjects were fitted with the sensors after arrival and completed a demographic data questionnaire afterward.

**Figure 5:**
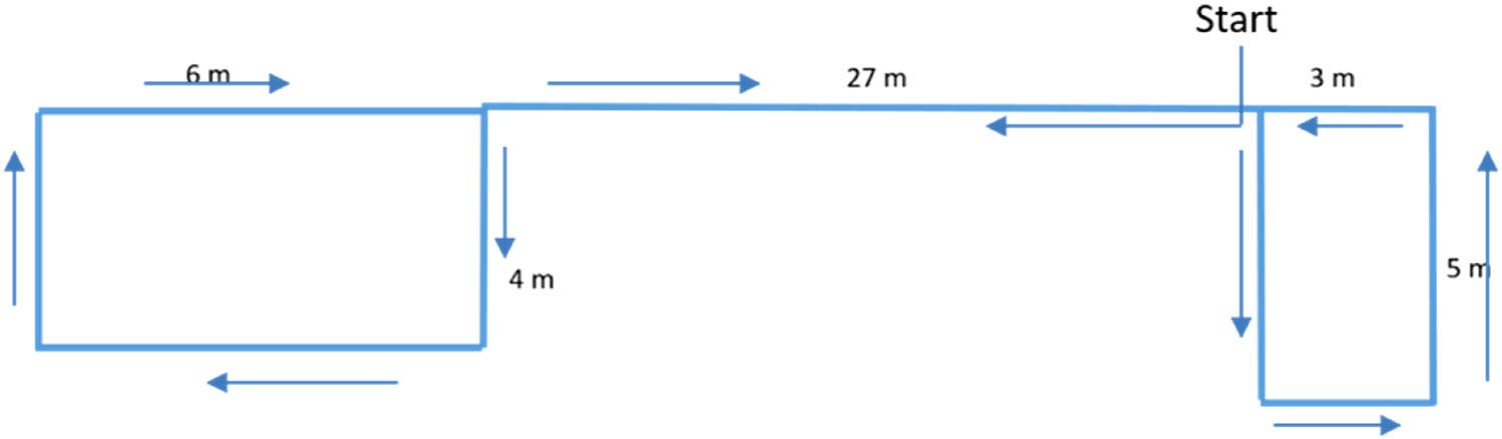
The course, which the subjects had to walk on their own and with the test leader.

For the walking task, a course of 90 m was set up (see Fig. 5). Before the first walking task, the experimenter showed the course to the subjects. Then the subjects walked the course alone while the EDA was measured. Also, the time was tracked to calculate the free walking speed. Subsequently, a baseline for the EDA was measured. For this, the subjects stood still on one spot for 3 min. For the “standardized” given speeds, a test leader walked ahead. The test leader set the pace using a metronome and floor markers with different distances. The experimenter traveled the distance between two floor markings in two beats of the metronome. The greater the distance, the faster the speed. The group of subjects walked behind each other at 1.5 m intervals. Overtaking was not allowed. The specified walking speeds were 1.41 m/s, 0.86 m/s, 0.31 m/s, and 0.19 m/s. The different speeds are taken out the fundamental diagram from Fruin^50^. While 1.41 m/s is slightly higher than density 0, the other speeds are connected to densities higher than 1, 2, and 3. Each speed was walked for 3 min. Afterward, the subjects filled in a questionnaire about their sensations during the experiments. The procedure was approved by the Ethics Council of the German Psychological Society (DGPs).

#### Questionnaire

**Figure 6:**
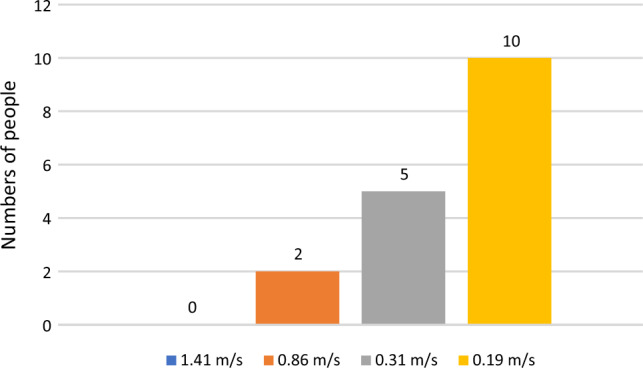
The figure shows the subjects’ subjective evaluation of which walking speed was perceived as most uncomfortable.

In addition to collecting demographic data, subjects were interviewed after the experiment. The questions can be found in Table 1. The questions referred to the experiences or sensations during the experiment. Responses were mapped using a 5-point Likert scale with “strongly disagree” and “strongly agree” poles (see Table 1). In addition, subjects who rated the very slow speed as unpleasant were asked what the reason was. Subjects also indicated which of the four given speeds was most unpleasant for them in terms of compliance (Fig. 6).

#### Skin conductance

The data were collected with a Movisens ambulatory system. Specifically, the EDA Move 4 was used^51^. The solidgel electrodes were placed on the palms of each subject’s non-dominant hand. The raw signal was collected at a sample rate of 32 Hz and was measured in micro-Siemens (µS).

Python (version3.7) was used to process the raw data. Preprocessing was based on the steps recommended in the literature^45,52^. The data was processed using a first order Butterworth low-pass filter with a cut-off frequency of 0.6 Hz to remove electrical noise from the data. For further analysis, the data were separated into the slowly changing tonic skin conductance level (SCL) and the quickly changing phasic skin conductance response (SCR)^53^. SCRs are classically responses that occur because of a stimulus, such as a startle event or an emotional stimulus. In laboratory experiments, the advantage is knowing when such stimuli were set and that the response occurs 1 to 5 s after the stimulus^27,28,54^. This kind of information about stimuli is not available in the context of studies in the field, but SCR is still considered as a parameter. This is because in addition to specific SCRs, there are spontaneously occurring SCRs (Ns.SCR) that have the same or similar structure as a specific SCR.

The separation of the data was done using the convex optimization approach of^55^ and was done for each experimental condition separately. Using the algorithm of^56^, the peaks (Ns.SCR) that had an amplitude greater than 0.01 µS were marked and then counted for each experimental condition. In addition to the Ns.SCR, the average amplitude height was also determined to detect differences in the strength of the stress responses. To calculate the parameter SCL, the mean value of the tonic component was used.

Since this is a study with motion, the data were controlled for artifacts. The control was done in two stages. First, a rule-based approach was used, oriented to the rules of Gashi et al.^45^, which flagged all rule violations in the data. In addition, the course of the accelerometer data collected as an indicator of physical activity was considered as in Gashi et al.^45^. This was followed by a visual inspection and the decision whether to use the data or not. Extreme swings in physical activity and the coincidence of a marked area in the data indicate an artifact due to movement and are excluded from further analysis^57^.

In addition to the classic parameters SCR and SCL, the parameter EDASymp was also used for evaluation. The parameter is based on a frequency dependent analysis and the idea behind the parameter is based on the analysis of heart-rate data. It is assumed that excitation associated with sympathetic activity is in a frequency range between 0.045 and 0.25 Hz^58^. For the parameter EDASymp, the raw data were processed analogous to the paper of Posada-Quintero and colleagues^59^. The data were down sampled to 2 Hz, followed by a fourier transform and power spectral analysis. For comparability of parameters between conditions, the area under the curve associated with sympathetic nerve activity was then calculated.

#### Analysis

The significant differences between the different experimental conditions were tested using one-way analysis of variance (ANOVA) with repeated measures. The parameters that were compared were EDASymp, Mean SCL, and the number of Ns.SCRs. The conditions were performed for 3 min; for the comparison of the freely chosen walking speed, the duration of the fastest subject was used to obtain time intervals of equal length. In addition, the total walked time of each experimental condition of 3 min was compared.

For the ANOVA test, normal distribution was checked using the Kolmorov-Smirnov test. Non-parametric tests were used for normal distribution violations. The individual conditions were then tested by a post hoc t-test using the Bonferroni method for multiple testing.

In addition to the differences examined between the individual given conditions of the subject group, the influence of the walking speed normally walked was also collected and examined in the condition “freely chosen walking speed.” For this purpose, the subjects were divided into two groups (fast and slow). All subjects walking faster than 1.39 m/s in the free walking speed condition were assigned to the fast group. The groups were examined for differences between the experimental conditions, and the differences between the groups fast and slow were also compared. For this, we used a mixed model in which speed was included as an independent variable and the grouping of the freely chosen speed as a mediator. A second mixed model was used to mediate the desire to overtake the front-runner on the parameters of the EDA. For comparisons between groups, the EDA data were z-standardized beforehand, orientated to Taylor and colleagues^44^. The mean and the standard deviation are the values from the baseline. Otherwise, they would not have been comparable due to the individual baseline values of the subjects^60^.

Also, the data of the questionnaire are considered descriptively. In addition, the data of the fast and the slow group are compared with each other with the help of t test. Furthermore, the effect size (Cohen's d) of the test is determined.

## Study 2

### Method

#### Participants

The study was conducted as part of a series of large-scale experiments on pedestrian dynamics in the Croma project. For more detailed information about the conducted sub-experiments see Boomers et al.^61^. For the study, 80 people were selected based on the criteria of age (< 35 years) and good neurological and physical health (appraised using self-reporting questionnaire). The gender distribution was 50%-50%. Of the 80 experimental participants, 56 subjects were fitted with sensors for measuring physiological arousal. After checking the EDA signals for artifacts such as null lines or extreme fluctuations due to movement or contact problems of the electrodes or incomplete data sets for each experimental condition, we excluded 12 participants from further analysis. The mean age of the participants included in the final analysis was 26 years (+ /−4). The final gender distribution of persons wearing EDA was 24 (54.5%) female and 20 (45.5%) male. The genders were evenly distributed in each experimental run, meaning that there was always the same number of female and male in the oval. The methods were following the Declaration of Helsinki.

#### Procedure and experimental paradigm

**Figure 7:**
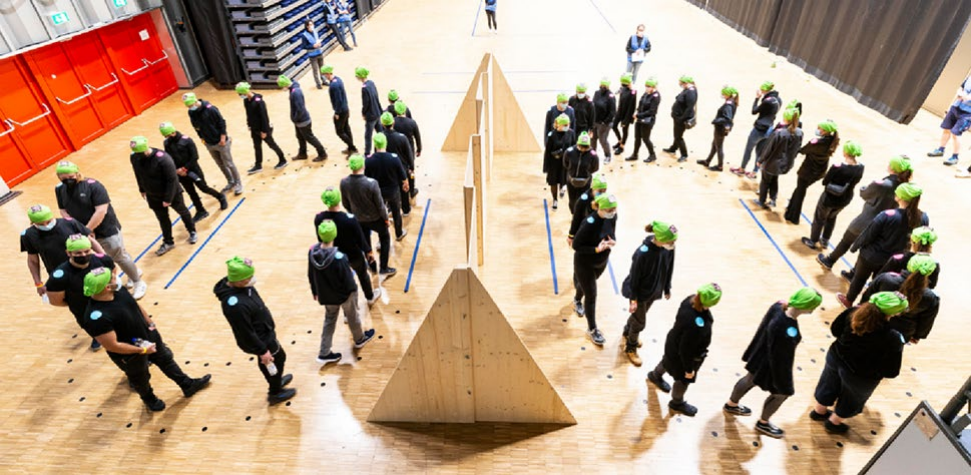
The picture shows the experimental setup and the execution of the experimental condition “20 persons per oval”.

The study was conducted in the Mitsubishi Electric Hall in Düsseldorf, Germany, in the context of large-scale experiments. Informed consent was obtained at the beginning of the experiments. The subjects could refuse to participate in individual experiments after being informed about the procedure of the experiment. The experiments were approved by the Ethics Council of the DGPs. A classical single-file experiment was used for this purpose^48^. Two ovals were marked on the floor, the circumference was 14.97 m with a walking width of 0.8 m (Fig. 7).

The ovals were separated from each other with a wooden wall. Each subject went through each experimental condition, but not necessarily in the same order. The different conditions are indicated in the table, together with the area available to each subject, excluding their own body. Also shown is the amount of space between subjects when the subjects are evenly spaced. For this purpose, the average body depth of 28.8 cm according to Buchmüller and Weidmann^62^ was subtracted from the available area (Table 10). In study 2, no questionnaire was used. However, at the end of the day, after the participants had finished several experiments, they filled out a questionnaire asking them to indicate how concerned they were about contracting Covid-19. The questionnaire also asked what other factors stressed and strained the participants in the experiments.

**Table 10:** Table showing experiment runs which the subjects went through at least once.

Number of persons in the oval	Space for each person in m	Space for each person after subtracting the depth of the human body in m
4	3.74	3.45
8	1.87	1.58
16	0.94	0.65
20	0.75	0.46
24	0.62	0.33
32	0.47	0.18
36	0.42	0.13
40	0.37	0.08

#### Skin conductance

The skin conductance data were again collected using Movisens ambulatory systems^51^. The raw data was processed according to the steps outlined for study 1. In addition, the same parameters were collected as in study 1.

#### Analysis

Possible significant differences between the different experimental conditions were tested using one-way analysis of variance (ANOVA) with repeated measures. The parameters that were compared were EDASymp, Mean SCL, and the number of Ns.SCRs (see study 1). The conditions were collected at intervals of different lengths. The shortest interval was used as a basis for the comparisons, and then the corresponding parameters of EDA for this period were determined.

Further analysis of gender was not performed after a study by Paetzke and colleagues^63^ using the same data set and additional experimental runs showed that gender played little or no role in oval speed or spacing. Moreover, such an analysis is laborious, since not only the gender of the person but also that of his or her predecessors and successors must be taken into account. The number of EDA gauges has not been sufficient to generate a dataset containing sufficient data points from each possible combination.

For the ANOVA tests, normal distribution was checked using the Kolmorov-Smirnov test. Then the individual conditions were tested by post hoc t-test using the Bonferroni method for multiple testing. If the assumption of sphericity was violated, the Greenhouse-Greisser correction was used.

### Supplementary Information

Supplementary Tables.

## Data Availability

The data used to support the findings of the manuscript are available from the corresponding author (Mira Beermann (mira.beermann@rub.de)) upon request.
