# Supplementary material for: The connection between stress, density, and speed in crowds
Source: Sci Rep. 2023 Aug 21;13:13626. doi: 10.1038/s41598-023-39006-8 (PMC10442413; doi:10.1038/s41598-023-39006-8)
Supplement: Supplementary file 1 — Supplementary Tables. [file 41598_2023_39006_MOESM1_ESM.docx]

| VP | Open question |
| --- | --- |
| 1 | I felt like we were barely making progress and it was a waste of time to go so slow |
| 2 | because it was much slower than I was used to and I had a hard time coordinating my body (legs & feet) |
| 3 | I felt unsteady and had a forward momentum. Also, my balance has wobbled and the steps have become unsteady |
| 4 | My normal walking pace is quite brisk, so it annoys me when I can't pass someone |
| 5 | comfortable, no feeling of being overwhelmed |
| 6 | Lead weight. Maintain distance. stop and go |
| 7 |  |
| 8 | Because I was used to the faster speeds and the very slow speed made me feel like I was wasting time. |
| 9 |  |
| 10 |  |
| 11 | Normally I run at a higher speed, so it was quite difficult for me to adapt to it. |
| 12 |  |
| 13 | I had to concentrate extremely hard not to overtake my predecessor. |
| 14 | I felt like an old grandpa there |
| 15 |  |
| 16 |  |
| 17 | Need to walk faster, feeling of not moving from the spot |

Table S1: Answers to the open question of why the slowest speed (0.19 m/s) was the most uncomfortable in Study 1.

| Parameter | β | t | p |
| --- | --- | --- | --- |
| Intercept | 23.02 | 3.241 | 0.002 |
| Speed | 1.80 | 0.75 | 0.48 |
| Desire to overtake | 0.31 | 0.14 | 0.89 |
| Speed: Desire to overtake | -0.35 | -0.46 | 0.65 |

Table S2: The model for the NS.SCR and the mediator desire to overtake.

| Parameter | β | t | p |
| --- | --- | --- | --- |
| Intercept | 26.94 | 5.74 | .001 |
| Speed | -1.33 | -.79 | 0.432 |
| Slow freely chosen walking speed | -5.72 | -.86 | 0.390 |
| Speed: Slow freely chosen walking speed | 3.98 | 1.73 | 0.089 |

Table S3: The model for the NS.SCR and the mediator slow freely chosen walking speed.

| Parameter | Β | t | p |
| --- | --- | --- | --- |
| Intercept | .0011 | .91 | .367 |
| Speed | -.00006 | -.20 | .842 |
| Desire to overtake | .0002 | .56 | .584 |
| Speed: Desire to overtake | -.00005 | -.51 | .615 |

Table S4: The model for the amplitude and the mediator desire to overtake.

| Parameter | Β | t | p |
| --- | --- | --- | --- |
| Intercept | .001 | 1.55 | .126 |
| Speed | .00002 | -.11 | .908 |
| Slow freely chosen walking speed | .0008 | .71 | .490 |
| Speed: Slow freely chosen walking speed | -.0004 | -1.49 | .143 |

Table S5: The model for the amplitude and the mediator slow freely chosen walking speed.

| Parameter | Β | t | p |
| --- | --- | --- | --- |
| Intercept | .033 | 2.43 | .019 |
| Speed | -.004 | -1.03 | .310 |
| Desire to overtake | .005 | -1.11 | .282 |
| Speed: Desire to overtake | . 0008 | .70 | .486 |

Table S6: The model for the EDASymp and the mediator desire to overtake.

| Parameter | Β | t | p |
| --- | --- | --- | --- |
| Intercept | .013 | 1.42 | .161 |
| Speed | .0006 | .26 | .780 |
| Slow freely chosen walking speed | .012 | .93 | .365 |
| Speed: Slow freely chosen walking speed | -.004 | -1.18 | .246 |

Table S7: The model for the EDASymp and the mediator slow freely chosen walking speed.
